# Emergency presentation of the gastric cancer; prognosis and implications for service planning

**DOI:** 10.1186/1749-7922-7-31

**Published:** 2012-09-25

**Authors:** Peter Vasas, Tom Wiggins, Asif Chaudry, Catherine Bryant, Frances S Hughes

**Affiliations:** 1 Academic Surgical Department, Barts and the London NHS Trust, Whitechapel, London (E1 1BB), United Kingdom

## Abstract

**Aims:**

To compare emergency and elective presentation of gastric cancer by mode of clinical presentation, initial stage, intervention and prognosis.

**Methods:**

Data were collected prospectively for all cases of gastric cancer presenting to a tertiary referral centre between 2003 and 2010. This was stratified by emergency and elective presentation and was analysed for mode of presentation, initial stage and outcome. Statistical analysis was performed using unpaired t-test and Chi^2^ test.

**Results:**

A total of 291 patients presented: Forty-two (14.43%) were emergencies and 249 (85.57%) elective presentations. Analysis of the emergency cohort showed 25 patients presented with obstruction (59.52%), 15 presented with haematemesis (35.71%) and 2 with perforation (4.76%).

Eighteen of the emergency patients (45%) presented with stage 4 disease compared to 60 (25.42%) in the elective group (p < 0.005). Fourteen of the emergency patients were treated with curative intent (33.3%) compared with 130 (55.56%) in the elective group (p < 0.01). Over 6 years only 2 patients needed operation within 24 hours of presentation.

Overall survival at one year for emergency patients was 48.3% compared to 63.4% in elective patients (p < 0.05). There were no survivors from the emergency group after 3 years but 32.46% of the elective patients survived (p < 0.02). Elective presentation with disease stage 1A-3B had a two year survival rate of 54.95% compared to only 20% in the emergency group (p < 0.05). Of patients who underwent operative intervention 67.44% of patients who presented electively survived to 2 years. This compared to just 25% presenting as emergencies (p < 0.001).

**Conclusions:**

Emergency presentation of gastric cancer is rare; is associated with higher stage of disease at presentation and lower rates of operability. The necessity to perform emergency operation within 24 hours is exceedingly rare. Emergency presentation is a marker of poor long term outcome for equivalent cancer stage in non-advanced (stages 1A-3B) disease.

## Introduction

Gastric cancer is the second most common cause of cancer death worldwide [1], being responsible for 650 000 deaths annually. In the UK in 2007, there were 5,236 deaths from stomach cancer, making it the seventh most common cause of cancer death and responsible for over 3% of all cancer related mortality [2]. In 2007 the age-standardised rate of gastric carcinoma in the UK was 5.7 per 100 000 population.

The majority of the patients present with non-acute symptoms but gastric cancer can also manifest as an emergency with haematemesis, visceral perforation, or gastric outlet obstruction. Emergency presentation of gastric cancer has been shown to have an influence on overall survival, which is independent to any other factors. Blackshaw et al. [3] showed that patients presenting as an emergency had a median survival of 6 months, compared to 12 months for patients referred as an outpatient. Therefore, although emergency presentation is relatively rare, it may significantly affect prognosis.

Recent advances in diagnostic tools and new oncological treatments may improve the overall outcome of gastric carcinoma, but emergency presentation continues to be associated with higher stage of disease at presentation and lower rates of operability. The majority of the peer-reviewed papers report 10-25 patients in the emergency group [4-7].

Perforated gastric cancer is rare accounting for 0.3-3% of gastric cancer cases [6-8], but gastric cancer is present in 10-16% of patients presenting with gastric perforation [9]. Only one-third of cases of perforated gastric cancer are diagnosed pre-operatively [7]. The diagnosis of gastric cancer is usually confirmed by post-operative histological examination. A two-staged procedural approach is sometimes used for the treatment of perforated gastric carcinoma; the first procedure controls the perforation and treats peritonitis, followed by a second procedure involving definitive gastrectomy with appropriate lymph node dissection [10,11].

Minor bleeding is a well-known characteristic of gastric cancer, often causing chronic microcytic hypochromic anaemia, prompting gastroscopy. However, gastric cancer can also present with major bleeding in up to 5% of patients [12]. These patients may require blood transfusion to prevent haemodynamic compromise. Endoscopic therapy can be used to control bleeding with the use of injection of adrenaline to the tumour base, argon plasma coagulation or with application of endo-clips [13]. However patients may require surgery for bleeding control if endoscopic measures for haemostasis fail.

Gastric outlet obstruction is more common than other emergency presentations and is usually a sign of locally advanced incurable disease. Traditionally, surgical bypass with gastrojejunostomy or palliative distal gastrectomy were the only therapeutic options to restore the gastric outflow. However increasingly, endoscopic stenting is utilised for to relieve obstruction in gastric cancer [14].

With specialist oesophagogastric surgeons being increasingly based in tertiary referral centres, there have been concerns that specialist surgeons may not be available should emergency surgical intervention be necessary in cases of gastric cancer. This raises the question of how commonly specialist oesophagogastric intervention is necessary in the emergency setting and how hospitals should plan their surgical service.

## Aims

This study aims to compare the influence mode of presentation (emergency or elective) has on the outcome of patients with gastric cancer in a deprived inner city area. The frequency with which emergency operative intervention within 24 hours of presentation is necessary will also be established. The study aims to provide suggestion for the service planning; as examine the surgeons sub-specialty training who were involved into the emergency operations.

## Patients and methods

Data were collected prospectively from all consecutive cases of gastric cancer patients presenting to the Upper Gastro-Intestinal Multidisciplinary Team at The Royal London Hospital between September 2003 and January 2010. Patient demographics, mode of presentation, disease stage at presentation, interventions and treatment undertaken, complications, hospital stay and survival were retrospectively analysed from the Departmental Database.

All consecutive patients presenting with gastric cancer to The Royal London Hospital or referred for treatment from one of the local diagnostic centres were involved. All of them were discussed at the specialised Multidisciplinary Team meeting; patients requiring urgent intervention often were discussed after initiation of treatment. Patients with stage IV disease or those deemed unfit for resection were diverted to a palliative care pathway. Fit patients with resectable disease were treated with curative intent. Neoadjuvant chemotherapy was considered in all patients with T3 or higher stage of cancer (according to the MAGIC trial) [15].

Emergency presentation was defined as those patients whom required immediate admission for treatment of symptoms (bleeding, perforation or obstruction). Major bleeding was characterised by the requirement of one or more unit of blood transfusion for acute blood loss.

Patients with cancer at the gastro-oesophageal junction were excluded, as were any patients undergoing prophylactic gastrectomy due to hereditary risk of gastric carcinoma.

Data was analysed to investigate the effect of emergency presentation upon the stage of disease at presentation and the proportion of patients treated with curative intent. The number of patients requiring emergency surgical intervention within 24 hours of presentation was recorded. Cumulative survival periods were calculated using the Kaplan-Meier method and differences in survival rates by disease stage were analyzed by COX-regression analysis. Comparison between the emergency and the elective presentations the χ^2^ test and Fisher’s exact test were used.

## Results

### Patient demographics and presentation

A total of 291 patients presented to our centre with gastric carcinoma during the 77-month period. Forty-two (14.4%) of these patients presented as an emergency with upper gastrointestinal (GI) bleeding, gastric perforation or gastric outlet obstruction. The remaining 249 patients (85.6%) presented electively via an outpatient referral with non-acute symptoms.

The mean age at presentation was 67 years in the emergency group and 68 in the elective group. From the emergency group twenty-five patients presented with obstruction (59.6%), two patients with perforation (4.8%) and 15 patients presented with upper GI bleeding (35.7%) and 7 of these patients required blood transfusion.

Elective patients presented with lower stage disease, stages 1 and 2 accounting for 37.6% of cases, compared with 23.1% of the emergency cases (p < 0.05). Twenty-five percent of elective cases presented with stage 4 disease, compared to 45% of the emergency cases (p<0.005).

**Figure 1:**
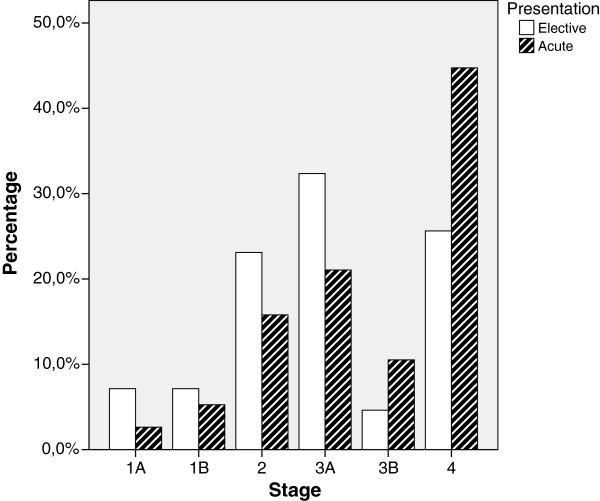
Stage at presentation.

### Interventions and operative procedures

One hundred sixty-nine patients underwent operative intervention (58.1%), the remaining 122 patients had oncological, endoscopic or supportive palliative care.

In the elective group 139 patients out of 249 (55.8%) were treated with curative intent, compared with 15 out of 42 (35.7%) in the emergency group (P < 0.05 with χ^2^ test).

In the emergency group 13 patients (30.9%) were unfit for any operative intervention and were treated palliatively, 14 patients (33.3%) underwent non-curative procedures (laparotomy with further procedure abandoned due to evidence of malignant spread (n = 3), gastro-jejunostomy (n = 6) or non-curative distal gastrectomy (n = 5)). Of emergency cohort patients 11 patients were suitable to undergo distal gastrectomy (26.2%) and total gastrectomy was performed in 4 cases (9.5%).

In the elective group the pre-operative assessment, cross-sectional imaging and laparoscopy identified 106 patients, (42.5%) with unresectable or metastatic disease or patients were unfit to undergo major surgery. A further 9 patients (3.8%) were found to be unresectable at operation, one of these patients underwent local excision. Three patients from the elective group who were suitable for resection declined the operative procedure. The surgical procedures performed are shown in Table 1.

**Table 1:** Operations performed

**N = 291**		**Presentation**			
		**Elective**		**Acute**	
		**Number of patients**	**%**	**Number of patients**	**%**
Type of operation	None	109	37.5	13	30.9
	Total gastrectomy	61	20.9	4	9.5
	Distal gastrectomy	69	23.7	16	38
	Gastro-jejunostomy	1	0.3	6	14.3
	Laparotomy/laparoscopy	8	2.7	3	7.1
	Local excision	1	0.3	0	0
				**Total**	**249**		**42**	

Inpatient stay for patients undergoing operative intervention was similar for both groups. The median post-operative hospital stay for the emergency group was 9.5 days (IQR = 4), compared to 12 days (IQR = 7) in the elective group.

#### Emergency surgery in the first 24 hours

Three patients required emergency operation within 24 hours of admission. This represents 1% of all presentations, and 7.1% of emergency presentations of gastric carcinoma. In each of these cases the emergency procedure was performed by the On-call General Surgeon (Breast, Colorectal and Hepato-Biliary specialists).

Two patients presented with gastric perforation and underwent emergency laparotomy. One patient was found to have metastatic disease and a palliative distal gastrectomy was performed. The second patient had a perforated gastric ulcer which was biopsied and an omental plug applied. The patient received palliative chemotherapy with no response. Survival for these two patients was 5 and 4 months respectively.

The third patient requiring emergency surgery presented with haematemesis to one of our local District General Hospitals. Although endoscopy confirmed a bleeding gastric ulcer, the haemorrhage could not be controlled endoscopically. The patient proceeded to theatre for laparotomy and a 3 cm ulcer high on the greater curvature was found with a central bleeding vessel. This was under-run and biopsies taken which confirmed adenocarcinoma. The patient made a good recovery and was referred to our centre for definitive oncological management. A total gastrectomy was performed six weeks following his initial presentation, the final histology was T1N0 adenocarcinoma, 0/39 nodes. The patient survived for two years following this procedure.

#### Emergency procedures after 24 hours

The remaining 39 emergency patients were managed without operative intervention over the first 24 hours. Fifteen patients presented with haematemesis. Nine received endoscopic intervention (injection, Argon-beam laser, heater probe) for bleeding control. Four patients were not actively bleeding at the time of endoscopy, and no further procedure was performed at this time. One patient had a large bleeding polyp removed at endoscopy, and three patients required injection of adrenaline to bleeding ulcerated areas. In one of these patients an endoclip was applied and argon plasma coagulation (APC) successfully performed. In only one case was endoscopic therapy not successful in controlling bleeding and this patient proceeded to theatre as described above. Overall 29 patients had some form of operation after complete staging, often on separate admission.

Patients presenting with gastric outlet obstruction were managed conservatively via nasogastric decompression in the initial period whilst further investigations were undertaken to stage their disease and plan further intervention. In 2 cases expanding metal stents were inserted endoscopically allowing oral intake and palliative oncological therapies.

Subsequently 3 out of 42 emergency patients (7.1%) and 44 out of 249 elective patients (17.6%) had neoadjuvant chemotherapy after their initial assessment (p < 0.05).

### Survival

#### Overall survival

Twelve patients from the elective group and three patients from the emergency were lost to follow-up.

**Figure 2:**
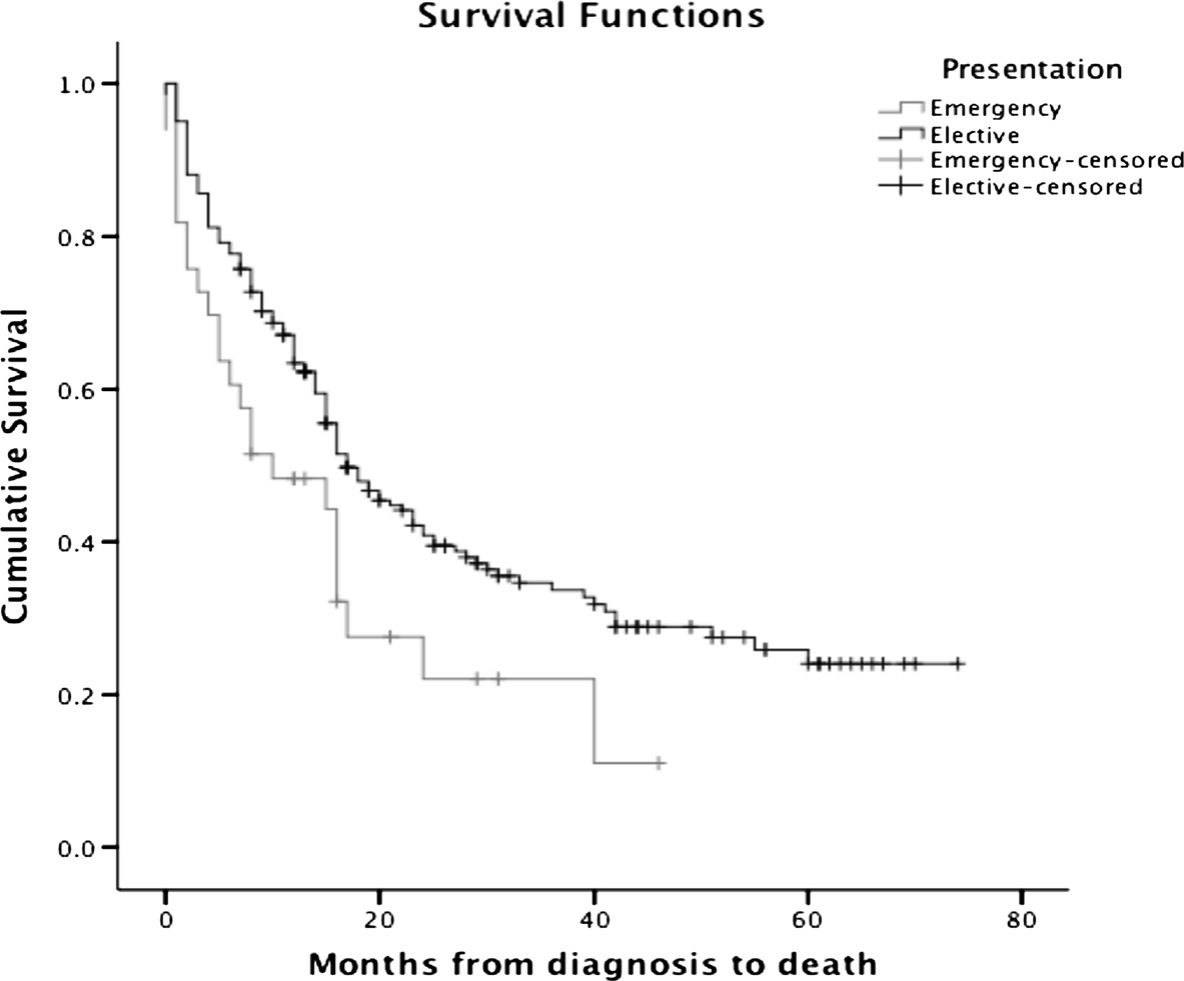
Kaplan-Meier curve showing comparison of survival between patients presenting as an emergency and electively.

One year survival for patients presenting as an emergency was 48.3% compared to 63.4% in elective patients (p = <0.02). By 3 years follow-up there were only two survivors from the emergency presentation group (14.3%), while 32.5% of the elective patients survived to 3 years (p = <0.006). The overall survival is shown on the Kaplan Meier plot on Figure 2.

#### Survival with non-metastatic disease

To examine survival for patients with comparable disease stage between the emergency and elective cohorts, all patients presenting with disease stage 1A-3B were further analysed. In the emergency group twenty-four patients (57.1%) presented with non-metastatic disease and the two year survival rate was 20.0% compared with 54.9% from elective group (189/249 patients). None of the emergency patients were alive after 40 months, while 36% of the elective group were alive at this stage.

**Figure 3:**
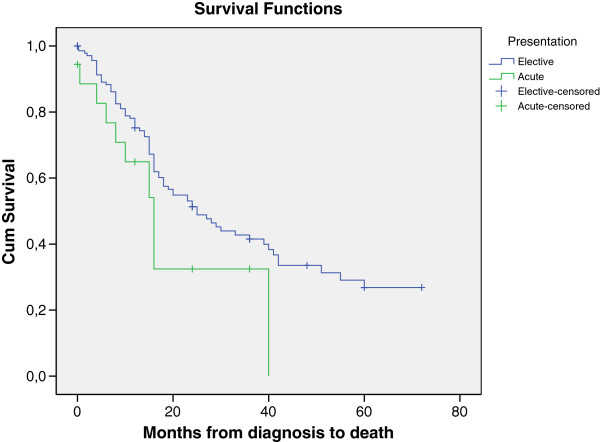
Comparison of survival for patients presenting with disease stage 1A-3B in the emergency and elective presentation groups.

The survival of patients with non-metastatic disease is shown in Figure 3.

#### Survival following curative resections

**Figure 4:**
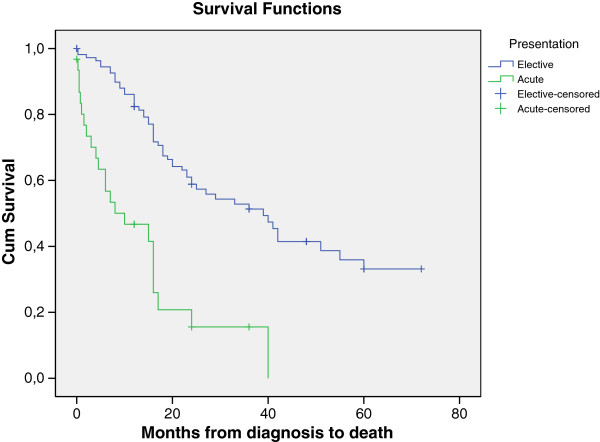
Comparison of survival for patients undergoing operative intervention in the emergency and elective presentation groups.

Of patients presenting as emergency who underwent subsequent resection 25% survived to 2 years. This compared to 67.4% two-year survival from elective group (p = <0.01). Five-year survival for elective patients undergoing operative intervention was 33.3% and there were no survivors in the emergency presentation group after 4 years (Figure 4).

## Discussion

Studies have shown that emergency presentation of gastric cancer is associated with higher stage disease and is an independent marker of poor prognosis. [3] Our results reinforce this as emergency patients more often presented with advanced stage disease; 45.0% of emergency patients presenting with stage IV, compared to 25.3% of elective patients (p <0.005), (Figure 1). Only 33.3% of emergency patients had resectable disease (compared to 55.6% of elective patients) (p <0.01). There were no survivors to 4 years follow up in the emergency group whereas 33.3% of operable elective patients survived to 5 years.

It is possible to claim that these results relate to the more advanced stage disease in the emergency group and not the presenting modality. However, when survival data for patients with non-metastatic gastric malignancy (stages 1A-3B) is analysed this shows that despite comparable disease stage, patients who present as an emergency have a worse prognosis and decreased survival. This may be due to the physical insult and the acute physiological deterioration during emergency presentation. Similar results were found when survival was compared for patients undergoing curative procedures. This suggests that emergency presentation could be an independent prognostic factor in gastric cancer. Other contributing factors to improved survival in the elective group may include the increased use of neo-adjuvant chemotherapy, and that patients presenting as an emergency may also be more severely malnourished at time of presentation.

Our results showed that the need for operative intervention within 24 hours of presentation is rare with only 3 patients (<10% of the emergency presentation) during this six-year period requiring emergency surgery. Two of these cases were as a result of gastric perforation, and one was due to bleeding despite attempts to control this via endoscopic therapy. These findings correlate with those of Blackshaw et al, who analysed 116 emergency presentations of gastric cancer between 1995 and 2003, and showed that none of these patients required operative intervention within 24 hours of presentation [3].

Centralisation of specialist oesophago-gastric service provision within tertiary referral centres has lead to many District General Hospitals losing their provision for specialist Oesophago-Gastric Surgeons on call. However as shown in this study the need for operative intervention within 24 hours of presentation of gastric carcinoma is exceedingly rare. In only one instance during this six-year series did endoscopic treatment fail to achieve haemostasis. This bleeding ulcer was successfully under-run at a peripheral hospital prior to definitive gastrectomy at our centre once the diagnosis of adenocarcinoma had been confirmed. Perforation of gastric cancer is also rare with a reported incidence rate of 0.3-3% of all cases of gastric carcinoma [6-8].

Performing gastrectomy in the context of gastric perforation and peritonitis presents numerous challenges. Inflammatory changes following peritonitis have lead to reported intra-operative overestimation of local tumour infiltration and lymph node involvement. [9] Therefore a two-staged approach to dealing with perforated gastric cancer has been proposed as the most suitable method. Lehnert et al recommend that the initial procedure should be directed at the treatment of perforation and peritonitis [9]. This involves either direct closure of the perforation or omental patch application, followed by thorough washout of the peritoneal cavity and drain insertion. Following patient recovery and histological confirmation of malignancy, accurate disease staging can be completed, and a radical oncological operation for gastric cancer or neoadjuvant chemotherapy can be planned as appropriate.

The initial emergency procedure should aim to simply control perforation and relieve peritonitis. Surgeons who are not specialists in Oesophago-gastric surgery could perform this initial procedure and the surgical training should address this question. The period of patient recovery following this emergency intervention would allow transfer to a tertiary referral centre for further assessment and management. Definitive gastrectomy can then be planned where appropriate. This period of planning for radical oncological intervention also allows time for patient optimisation, including nutritional support where necessary. Patients with gastric malignancy are often severely malnourished and a period of pre-operative nutritional optimisation, which is continued post-operatively may reduce complication rates [10].

## Conclusion

Emergency surgery within 24 hours of presentation for gastric malignancies is extremely rare. A two-stage approach for management of perforated gastric carcinoma could provide acceptable results and allows patients to be transferred to a tertiary Oesophago-Gastric centre for further assessment prior to definitive treatment; however our observation based on the limited patient number. Our experience shows that emergency lifesaving intervention can be successfully followed by transfer for emergency cancer therapy with reasonable survival. Emergency presentation is usually associated with advanced disease stage and resources should be diverted towards early diagnosis, increasing patient awareness rather than upper GI surgical services on all District General Hospital site.

## Competing interests

The authors declare that they have no competing interest.

## Authors’ contribution

PV: data collection, analysis and conclusions; TW, AC, CB: data collection and processing, FH: study design, paper review. All authors read and approved the final manuscript.
